# Controlled Growth and the Maintenance of Human Pluripotent Stem Cells by Cultivation with Defined Medium on Extracellular Matrix-Coated Micropatterned Dishes

**DOI:** 10.1371/journal.pone.0129855

**Published:** 2015-06-26

**Authors:** Chiemi Takenaka, Hiroshi Miyajima, Yusuke Yoda, Hideo Imazato, Takako Yamamoto, Shinichi Gomi, Yasuhiro Ohshima, Kenichi Kagawa, Tetsuji Sasaki, Shin Kawamata

**Affiliations:** 1 R&D Center for Cell Therapy, Foundation for Biomedical Research and Innovation, TRI#308, 1-5-4 Minatojima-Minamimachi, Chuo-ku, Kobe, Hyogo 650-0043, Japan; 2 Product Development Division, Kyokuto Pharmaceutical Industrial Co., Ltd., 3333-26, Aza-Asayama, Kamitezuna Takahagi-shi, Ibaraki, 318-0004, Japan; 3 Technology Development Center, Tokyo Electron Limited, Akasaka Biz Tower, 5-3-1 Akasaka, Minato-Ku, Tokyo, 107-6325, Japan; Instituto Butantan, BRAZIL

## Abstract

Here, we introduce a new serum-free defined medium (SPM) that supports the cultivation of human pluripotent stem cells (hPSCs) on recombinant human vitronectin-N (rhVNT-N)-coated dishes after seeding with either cell clumps or single cells. With this system, there was no need for an intervening sequential adaptation process after moving hPSCs from feeder layer-dependent conditions. We also introduce a micropatterned dish that was coated with extracellular matrix by photolithographic technology. This procedure allowed the cultivation of hPSCs on 199 individual rhVNT-N-coated small round spots (1 mm in diameter) on each 35-mm polystyrene dish (termed “patterned culture”), permitting the simultaneous formation of 199 uniform high-density small-sized colonies. This culture system supported controlled cell growth and maintenance of undifferentiated hPSCs better than dishes in which the entire surface was coated with rhVNT-N (termed “non-patterned cultures”). Non-patterned cultures produced variable, unrestricted cell proliferation with non-uniform cell growth and uneven densities in which we observed downregulated expression of some self-renewal-related markers. Comparative flow cytometric studies of the expression of pluripotency-related molecules SSEA-3 and TRA-1-60 in hPSCs from non-patterned cultures and patterned cultures supported this concept. Patterned cultures of hPSCs allowed sequential visual inspection of every hPSC colony, giving an address and number in patterned culture dishes. Several spots could be sampled for quality control tests of production batches, thereby permitting the monitoring of hPSCs in a single culture dish. Our new patterned culture system utilizing photolithography provides a robust, reproducible and controllable cell culture system and demonstrates technological advantages for the mass production of hPSCs with process quality control.

## Introduction

Cell therapy using human pluripotent stem cell (hPSC)-derived cells has been used for the treatment of several diseases. Applications include the use of human embryonic stem cell (hESC)-derived oligodendrocyte progenitor cells for the treatment of acute spinal injury [1] or hESC-derived retinal pigment epithelium (RPE) for the treatment of dry type age-related macular degeneration (AMD) [2]. Moreover, human induced pluripotent stem cell (hiPSC)-derived RPEs have been used for treatment of wet type AMD [3–6]. It is apparent that improved methods for culturing hPSCs are needed to meet regulatory demands ensuring the safety and quality of cellular products. For example, hPSCs can be maintained with animal component-free, chemically-defined and xeno-free media [7–10] under feeder-free conditions, e.g., cultivation in the presence of laminin-521, laminin-511 E8, pronectin, or rhVTN-N [11–14]. These synthetic peptides can be coated on dishes to permit feeder-free cultivation. Such peptides must be evaluated in terms of their potential for anchoring hPSCs in the culture medium as well as their cost. Among these synthetic peptides, rhVNT-N is competitive in price, costing 1–5% as much per dish as other available peptides. As for culture media, chemically-defined media without animal components would be ideal in terms of regulatory concerns, but several elaborate adaptation procedures are required in many cases (https://tools.lifetechnologies.com/content/sfs/manuals/feeder_free_hPSCs_in_essential8_medium.pdf, http://www.stemcell.com/~/media/Technical%20Resources/2/E/7/7/B/29267MAN.pdf?la=en) to adapt hPSCs on feeder layers to grow in a feeder-free environment (especially on rhVTN-N). In this report, we introduce a new medium and culture technique. The advantages of single cell patterning culture are discussed.

## Materials and Methods

All experiments using human cell lines and animals were reviewed and approved by the committee for non-clinical research and the committee for animal experimentation of the Foundation for Biomedical Research and Innovation (FBRI).

### Components of SPM medium

In this study, we used a newly developed product, SPM cell culture medium. This is a feeder-independent, serum-free, defined medium for hPSCs and is manufactured by Kyokuto Pharmaceutical Industrial, Japan. It contains 21 amino acids (L-alanine, L-arginine, L-asparagine, L-aspartic acid, L-cysteine, L-cystine, L-glutamic acid, L-glutamine, glycine, L-histidine, L-isoleucine, L-leucine, L-lysine, L-methionine, L-phenylalanine, L-proline, L-serine, L-threonine, L-tryptophan, L-tyrosine, and L-valine) as well as 12 vitamins (L-ascorbic acid, cobalamin, biotin, folic acid, I-inositol, niacinamide, d-calcium pantothenate, pyridoxine hydrochloride, riboflavin, thiamine hydrochloride, α-tocopherol and 4-aminobenzoic acid). It also includes trace elements, fatty acids, bovine serum albumin (BSA) and growth factors including 100 ng/mL bFGF (Peprotech, London, UK) at the time of preparation. It is stored at 4°C after thawing. The BSA was derived from New Zealand in compliance with the TSE guideline [15,16].

### Cell culture

The hESC lines KhES-1 (Riken BRC) [17], H9 (WiCell) [18] and the hiPSC lines PFX#9 [13] and 201B7 (Riken BRC) [6] were cultured and maintained on mitomycin C-treated SNL76/7 cells (SIM strain embryonic fibroblast, ECACC; European Collection of Cell Culture) with hPSCs culture media (DMEM/F-12; Sigma, Tokyo, Japan) containing 20% knockout serum replacement (KSR; Life Technologies, Carlsbad, CA, USA), 2 mM L-glutamine (Life Technologies), 1% non-essential amino acids (NEAA; Life Technologies), 0.1 mM 2-mercaptoethanol (Life Technologies) and 4 ng/mL bFGF. To transfer hPSCs cultured on feeder cells directly to feeder-free conditions, cells in cultures were treated for 5 min with CTK solution (phosphate-buffered saline; Life Technologies) containing 0.25% trypsin (Life Technologies), 1 mg/mL collagenase IV (Life Technologies), 20% KSR and 1 mM CaCl_2_. The resultant small cell clumps were cultured with SPM either on SNL76/7 at a split ratio of 1:3 for iPSC or 1:3.5 for hESC, or on rhVNT-N- (Life Technologies) coated dishes. Alternatively, hPSCs cultures on feeder layers were treated with CTK solution for 5 min followed by TrypLE Select (Life Technologies) for 5 min for dissociation and pipetted into single cells. The resultant single cells were cultured on rhVNT-N-coated non-patterned culture dishes or on rhVNT-N-coated patterned culture dishes at 37°C in a 5% CO_2_ atmosphere in an incubator (MCO-19AIC, Panasonic, Osaka, Japan). hPSCs were passaged with Gentle Cell Dissociation Reagent (GCDR; StemCell Technologies, Vancouver, CAN) for cell clump culture or TrypLE Select for single cell cultures.

### Cryopreservation and thawing procedures

hPSCs in single cell cultures were harvested with TrypLE Select and resuspended with chemically defined freezing medium (STEM-CELLBANKER; TakaraBio, Shiga, Japan) (1 x 10^6^ cells/mL) followed by transfer to cryovials (Iwaki, Tokyo, Japan) using a standard slow-freezing method. Cryovials were placed into a freezing container (Nalgene Cryo 100B0C Freezing Container, Nalgene, NY, USA) and cooled overnight in a -80°C freezer (MDF-U32V, Panasonic). Cells were then transferred to and stored in a -150°C freezer (MDF-1155AT, Panasonic) for at least 1 week before performing thawing experiments. For thawing, cryovials were warmed in a water bath at 37°C until the icy masses disappeared, and cell suspensions were transferred to 15 mL centrifuge tubes (BD Bioscience, MA, USA,) and diluted by addition of 4 mL SPM medium containing 10 μM Rock inhibitor Y-27632 (Nacalai Tesque, Kyoto, Japan). Cells were pelleted by centrifugation (120 x g, 3 min) and resuspended in 1 mL of fresh SPM medium containing 10 μM Y-27632 and seeded in 1 well of rhVTN-N-coated 6-well dishes as single cells and cultured at 37°C, 5% CO_2_. Media were changed every day.

### *In vitro* differentiation and teratoma formation assay

hPSCs were transferred to ultra-low attachment 6-well plates (Corning, NY, USA) to demonstrate their 3 germ layer differentiation potential by forming embryoid bodies (EBs). Cell clumps were incubated in the culture medium without bFGF and the medium was changed every three days. After 21 days of cultivation, EBs were transferred to 0.1% gelatin-coated 6-well plates. Cells were cultured for another 9 days with the same medium for differentiation, changing medium every three days. Expression of lineage-specific genes was examined by quantitative RT-PCR at the end of culture and cells were immunostained with antibodies for differentiation markers. For teratoma formation assays, 1 x 10^6^ hPSCs that had been cultured for 20 passages in SPM on rhVNT-N-coated dishes were embedded in 100 μL of Matrigel (BD Bioscience) and were transplanted under the epidermal space of the neck of NOD.Cg-*Prkdc* Il2rg/SzJ mice (Charles River, Kanagawa, Japan). Eight weeks after transplantation, mice developed teratomas at the injection site. Teratomas were fixed with 4% formalin (Wako, Kyoto, Japan) after extraction, and sliced sections were stained with hematoxylin (Sakura Finetek, Tokyo, Japan) and eosin (Merck Millipore, Darmstadt, Germany) for histological examination for 3 germ layer differentiation.

### Immunohistochemistry (IHC)

Differentiated cells from embryoid bodies were fixed on dishes with 4% PFA overnight at 4°C, then washed 3 times with PBS(-) for 5 min. Expression of α-SMA, β-tubulin or AFP in cultured cells was detected with the indicated antibody: anti-α-SMA antibody (1:200; V6630, Sigma, Tokyo, Japan); anti-β-tubulin antibody (1:100 T4026; SIGMA); anti-AFP antibody (1:100 MAB1368; R&D Systems, MN, USA). Binding was visualized with a secondary antibody labeled with Alexa Fluor 488 (1:500; A11008, Life Technologies) or Alexa Fluor 594 (1:500; A11062, Life Technologies), and cells were counterstained with DAPI (D1306, Life Technologies). An Olympus IX71 fluorescence microscope was used for fluorescent observation.

### Quantitative RT-PCR

Total RNA was extracted using an RNeasy micro kit (74004, QIAGEN, Hilden, Germany) according to the manufacturer’s instructions. One μg of total RNA was used to synthesize cDNA with the QuantiTect Reverse Transcription Kit (205311, QIAGEN). Quantitative PCR (qPCR) was performed with TaqMan hPSC Scorecard Panel (A15870, Life Technologies)[19], using a qRT-PCR device (QuantStudio 12K Flex, Life Technologies).

### Flow cytometric analysis

hPSCs were harvested and dissociated to single cells with TrypLE Select. The cells were washed once in a final concentration of 2% human serum albumin (Tanabe-Mitsubishi Pharma, Osaka Japan) in PBS(-). A total of 5 × 10^5^ cells was incubated with the same buffer containing 1/100 volume of Alexa Fluor 647-conjugated anti-SSEA-3 (BD, 561145) or BV421-conjugated anti-TRA-1-60 (562711, BD Bioscience) for 30 min at 4°C. The dead cells were distinguished with 7-amino-actinomycin D (7AAD, 559925, BD Bioscience). The cells were analyzed with a FACS Aria II cell sorter (BD Bioscience) and the data were analyzed using FlowJo software (FlowJo LLC, OR, USA).

### Karyotypic analysis

G band karyotyping service was provided by Nihon Gene Research Laboratories, Inc., (Sendai, Japan). Briefly, hiPSC were treated with colcemid (Sigma) and were harvested by treatment with 0.25% trypsin/EDTA. Cells were fixed on slides with Carnoy’s solution and soaked in Giemsa stain solution (Merck Millipore). After washing with water, 50 metaphase spreads were screened and 20 of them were evaluated for chromosomal rearrangements by microscopy (Eclipse E600, Nikon, Tokyo, Japan) at 1000x magnification for G-band analysis. For multi-color fluorescein *in situ* hybridization mFISH analysis, hPSCs fixed on glass slides were hybridized overnight with a 24XCyte mFISH probe kit (000000-0514-056; MetaSystems, MA, USA). Sections on glass slides were hybridized, and DAPI/anti-fade (000000-0542-060; MetaSystems) was applied per the manufacturer’s instructions for multi-color fluorescence *in situ* hybridization (mFISH) analysis. Metaphase cells were identified at 64x magnification using an Axio Imager.

### Micropatterning of culture dishes

Micropatterning of culture dishes was achieved by photolithographic technology as follows. The entire bottom of each polystyrene 35-mm dish (BD Bioscience) was coated with a water-soluble PVA-based, UV-sensitive polymer (BIOSURFINE-AWP, Toyo Gosei Co., Ltd., Tokyo Japan) that became insoluble after crosslinking with UV irradiation at 30 mW/cm^2^ for 1 sec with an MA6 mask aligner (SUSS MicroTec AG, Garching, Germany). The spots for cell culture were masked whereas the area not used for cell culture was not masked, permitting UV exposure. The masked area remained soluble and could be washed with Milli Q water. rhVTN-N was directly coated on the bottom of the polystyrene dish on the masked area by incubating 0.5 μg/cm^2^ of rhVTN-N for 1 h at room temperature just prior to use. rhVTN-N did not coat the unmasked insoluble PVA after UV irradiation. In this way, we were able to readily prepare rhVTN-N-coated micropatterned dishes.

## Results

### hPSCs cultured on feeder cells were readily adapted to feeder-free culture with SPM

**Fig 1:**
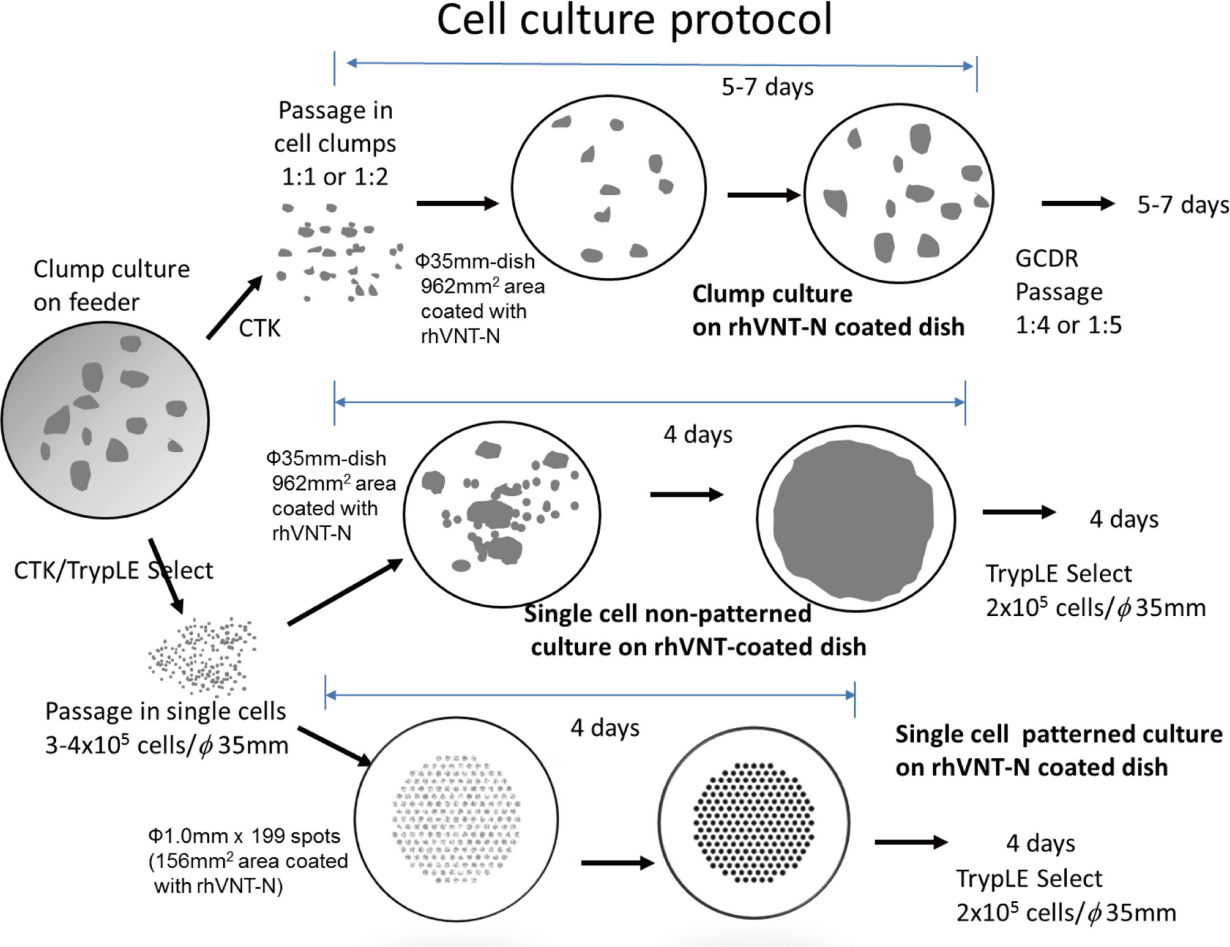
Cell culture protocols. (Top) hPSCs cultured in cell clumps on feeder cells (SNL) were harvested with CTK to culture in cell clumps on recombinant rhVTN-N-coated dishes with SPM medium by splitting at 1:1 or 1:2 ratios (clump culture). (Middle, Bottom) Alternatively, hPSCs were harvested with CTK followed by TrypLE Select to seed 3 or 4 x 10^5^ single cells on either rhVNT-N fully coated 35-mm dishes (single cell, non-patterned culture) or on rhVNT-N spot-coated 35-mm dishes (single cell patterned culture). hPSCs were cultured for 5 to 7 days and passaged at ratios of 1:4 or 1:5 splits with GCDR in cell clumps. hPSCs (2 x 10^5^ single cells) were seeded on each 35-mm dish for both non-patterned and patterned dishes, cultured for 4 days and dissociated with TrypLE Select.

hPSCs (hESCs/hiPSCs) cultured on SNL feeder cells were harvested either with CTK (clumps) or CTK followed by TrypLE Select (single cells). They were subsequently seeded on rhVNT-N-coated dishes. The first passage of cell clumps was split at ratios of 1:1 to 1:2 and cultured for 4 or 5 days. hPSCs must adapt to their new feeder-free environment and recover from the perturbation of cell growth after the first passage. Then, they were passaged at 1:4 or 1:5 with GCDR for subsequent passages (from the second passage onward). Cells (3–5 x 10^5^) in a single cell suspension were seeded either on 35-mm dishes (the entire surface was coated with rhVNT-N: non-patterned culture) or on 35-mm dishes micropatterned with 199 discrete spots (1 mm in diameter) coated with rhVNT-N (patterned culture) for the first passage and cultured for 4 days. Cells (2 x 10^5^) in a single cell suspension were seeded on either non-patterned dishes or patterned dishes and cultured for 4 days from the second passage onward as their proliferation potential stabilized during their first cultivation on rhVNT-N-coated dishes. TrypLE Select was used for dissociation. The culture protocol is shown in Fig 1. It is notable that SPM allowed hPSCs to be cultured directly from a feeder layer to feeder-free conditions (both patterned and non-patterned cultures) without a sequential adaptation procedure.

### Single cell culture supported the maintenance of hPSCs

**Fig 2:**
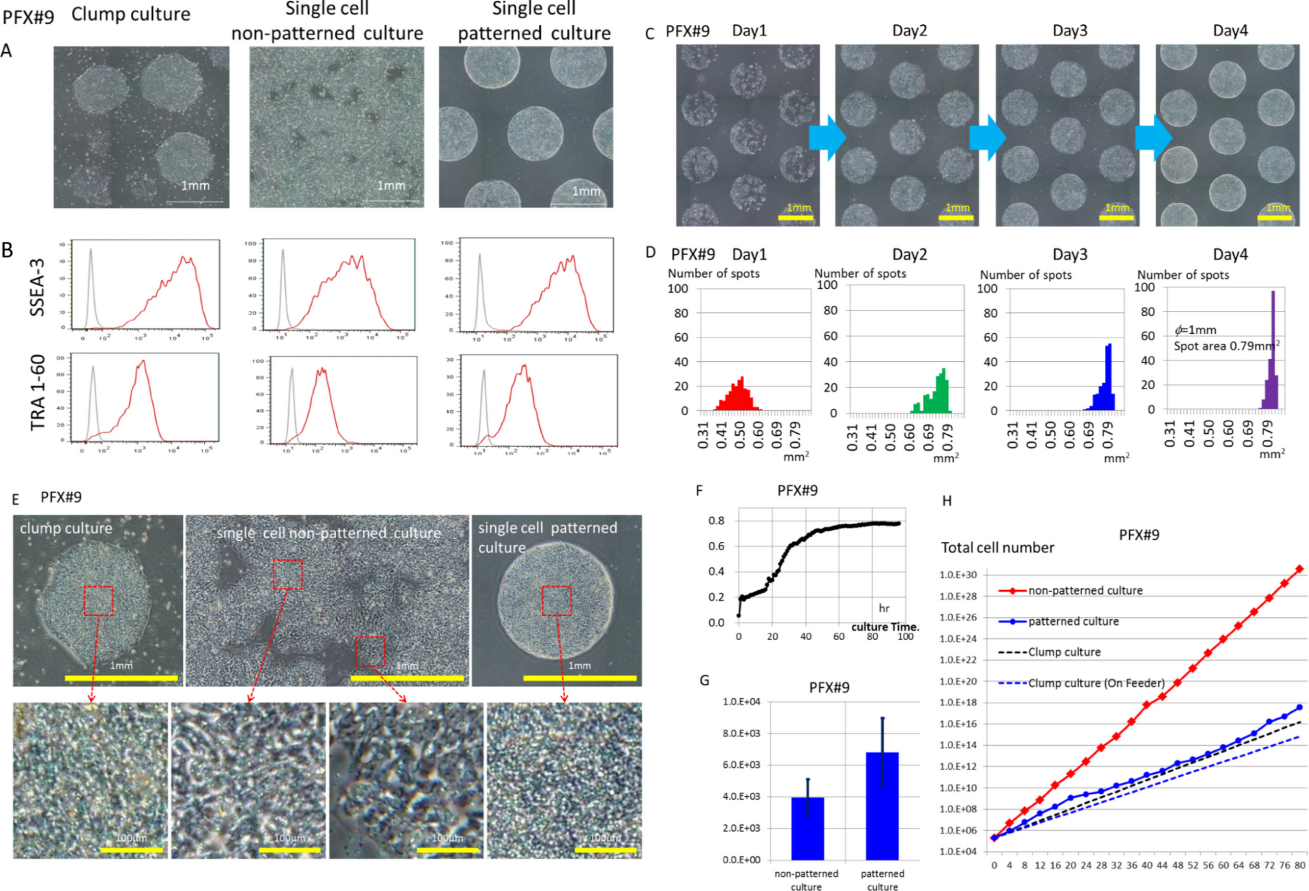
Culture of hPSCs with SPM on rhVNT-N coated dishes. (A) Phase contrast microscopic observation of iPSC cell line PFX#9 at passage 15. (B) Expression of SSEA-3 and TRA-1-60 by flow cytometric analysis of PFX#9 in indicated culture conditions at passage 15. (C) Time course of cell proliferation in patterned dish from days 1 to 4 (left to right). (D) Cell proliferation area that was occupied inrhVTN-N-coated spot area (spot Φ = 1 mm, 0.79 mm^2^/spot, X axis). Plot also shows the number of spots and their areas (out of 199 rhVNT-N-coated spots, Y axis) on days 1 to 4 (left to right). (E) Microscopic observations of clump cultures, single cell non-patterned or single cell patterned cultures with the higher magnified area in red rectangles at passage 20. A representative undifferentiated clump colony is shown in the upper left photo. Scale bars are appended. (F) Time course (0–100 h) of the area occupied by PFX#9 cells (in mm^2^) in 5 randomly selected spots (0.79 mm^2^/spot) measured by captured image analysis software (ImageJ 1.450, National Institutes of Health, Bethesda, MD, USA) every hour. Average of cell occupation area at every hour is plotted as a dot. The dot graph shows representative results from 3 independent trials. (G) Cell density (cells/mm^2^) of PFX#9 in single cell non-patterned or in single cell patterned culture was calculated by dividing harvested cell number by 962 mm^2^ (35-mm non-patterned culture dish) or dividing harvested cell number by 156 mm^2^ (total 199 spots of 1 mm diameter in 35-mm patterned culture dish). The results were obtained by scoring harvested cell numbers from 18 passages of indicated cultures and are shown as a bar (mean) with error bar (standard deviation). The significance of difference between 2 groups, p = 1.45 x 10^−9^. Representative results of 3 independent trials are shown. (H) Growth curve of PFX#9 in non-patterned culture, patterned culture or clump culture are shown in logarithmic graphs. PFX#9 cells in patterned culture or non-patterned culture were passaged every 4 days and in clump culture on feeder-free every 6 days and on feeder (SNL) every 5 days respectively.

**Fig 3:**
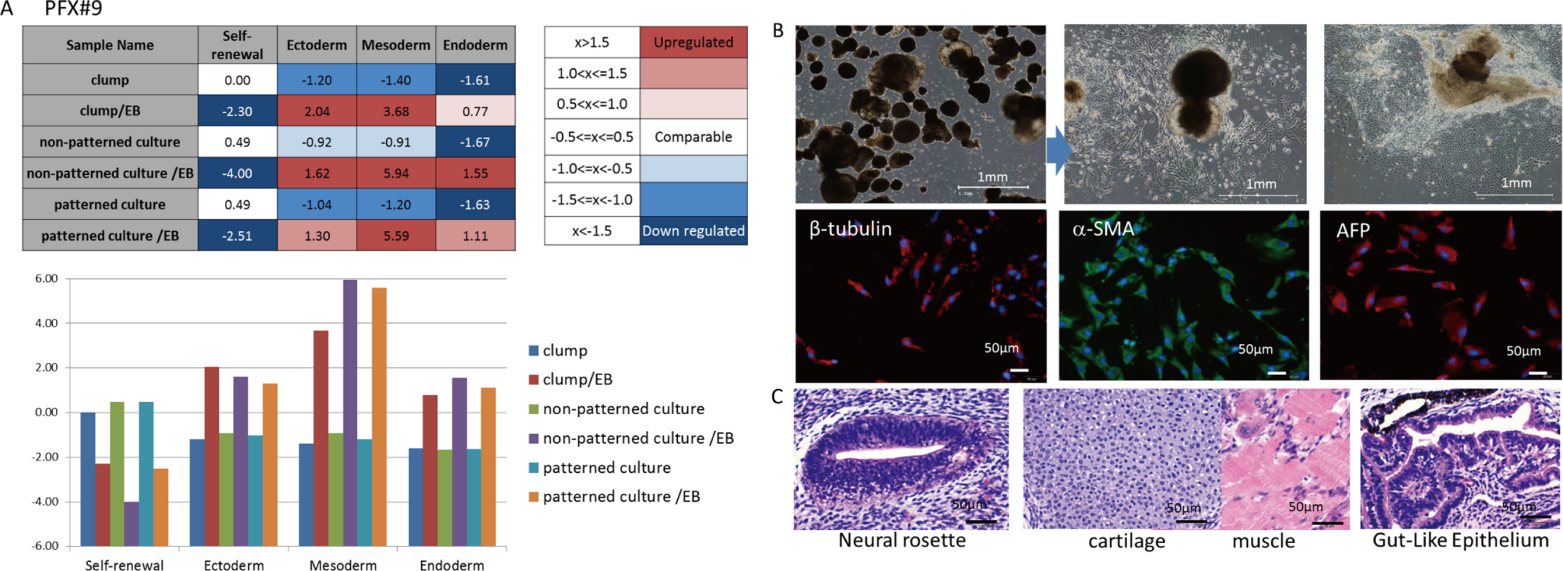
Differentiation potential of hPSCs. (A) Gene expression profiles of PFX#9 cells in the indicated culture condition (clump culture, single cell non-patterned culture or single cell patterned culture) before (undifferentiated state) and after induction of differentiation via embryoid body (EB) formation. Average of gene expression values for self-renewal (undifferentiated state), ectoderm-, mesoderm- or endoderm-related genes is shown in comparison with reference standards of TaqMan hPSC Scorecard Panel (Life Technology). (B) EB formation at day 14 from PFX#9 cells (top left). EB attached to culture dish and continued to differentiate (top right). Cells were then stained with antibodies against β-tubulin (ectoderm), α-SMA (mesoderm), AFP (endoderm) and DAPI. (C) Tissue section of teratoma in NOG mouse generated by inoculating PFX#9 cells maintained in cell clumps is shown after staining with HE. Three germ layers of tissue consisting of neural rosette (ectoderm), muscle/cartilage (mesoderm) and gut-like epithelium (endoderm) are observed. Scores in Tables are visualized in bar graph below.

Morphological observations and flow cytometric analyses showed that hiPSC cell lines PXF#9 and 201B7 and the hESC cell lines KhES-1 and H9 could be cultured in an undifferentiated state in three ways: as cell clumps or as single cells with SPM on rhVTN-N-coated dishes (non-patterned) or on rhVTN-N-coated spots in micropatterned dishes (patterned culture) (Fig 2A, 2B, 2C, 2D, 2E and 2F; S1A, S1B and S1C Fig). The SPM/rhVNT-N-coated dish culture system supported cell proliferation of hPSCs (Fig 2G and 2H, S1D, S1E, S1F and S1G Fig). Gene expression profiling of self-renewal-related genes determined by a qRT-PCR panel supported these data (Fig 3A, S3 Fig). Flow cytometric analysis and gene expression profiling of hPSCs in clump culture was conducted after removing differentiated colonies (cleaned colonies) on a daily basis. If differentiated colonies were not removed, the cell cultures generally committed most likely to ectodermal differentiation (S4 Fig). However, single cell seeding avoids this elaborate manipulation, as we examined the gene expression profile (S4 Fig) and self-renewal-related surface molecules just after harvest. Single cell culture supported greater yields of hPSCs at every passage (Fig 2H, S1E, S1F and S1G Fig) and facilitated quality control of hPSCs at the single cell level. Cultured and seeded hPSCs in a single cell suspension could be sorted for self-renewal-related surface markers to maintain an undifferentiated population during long-term culture if required (S2A Fig).

### Patterned cultures sustained controlled cell growth and the undifferentiated state of hPSCs

A greater yield of hPSCs was harvested from non-patterned cultures than from patterned cultures (Fig 2H, S1E, S1F and S1G Fig). In our micropatterned 35-mm dishes, the total area coated with rhVNT-N was 156 mm^2^, whereas that in non-patterned 35-mm dishes was 962 mm^2^. Whereas the coated area of the non-patterned dish was 6.16-fold greater, the number of cells harvested was only 4.23-fold greater than that from patterned cultures (Fig 2H). We harvested an average of 3.9 ± 0.81 X 10^6^ PXF#9 cells (n = 18) from non-patterned cultures and 9.2 ± 1.36 x 10^5^ PFX#9 cells (n = 18) from patterned cultures 4 days after seeding 2 x 10^5^ cells in both types of dishes. The number of cells harvested from a patterned dish could be increased by increasing the number of 1mm diameter spots and/or decresing the pitch between patterned spots. Nevertheless, our micropatterned 35-mm dish with a 156 mm^2^ rhVNT-N-coated area produced a comparable cell number with clump cultures in 35-mm dish (Fig 2H, S1E, S1F and S1G Fig). Some of the cells that were seeded on non-rhVNT-N-coated areas in patterned dishes could migrate to rhVNT-N coated areas, but the others could not survive because they were not anchored with rhVNT-N. Indeed, patterned culture restricted cell growth to the coated spots, producing uniform cell clumps (1mm in diameter) with small uniform cell sizes similar to cells in “good” clump cultures (Fig 2E, S1 Movie). In contrast, in non-patterned cultures, we observed uneven, unrestricted and amorphous spread of cell growth and disorganized orientation (Fig 2E). The number of harvested cells from non-patterned and patterned culture changed depending on when we harvested cells on “day 4”; thus, the calculated cell density may vary. The cell density of patterned culture was greater than that of non-patterned monolayer culture (Fig 2G: PFX#9, n = 18, p = 1.45 x 10^−9^; S1D Fig: KhES-1, n = 20, p = 3.6 x 10^−16^).

It is possible that uneven cell growth on non-patterned cultures as evidenced by distinctively different cell morphologies in the rim and in the center of the colony may not be favorable for the maintenance of the self-renewal potential of hPSCs as a population in long-term culture (Fig 2E). In contrast, patterned cultures in 1 mm diameter spots could support controlled cell growth and the maintenance of the undifferentiated state of hPSCs. We compared the expression of SSEA-3 and TRA-1-60 from non-patterned and patterned cultures of KhES-1 and PFX#9 by flow cytometry at passage 15 from the same population (Fig 2B, S1B Fig), and found higher expression of SSEA-3 and TRA-1-60 in patterned cultured than that in non-patterned culture. Further flow cytometric data of PFX#9 cells cultured in non-patterned dishes collected 5 passages after sorting showed downregulation of SSEA-3 and TRA-1-60 expression during passage (S2A Fig). These data supported this idea. The size of the patterned spots was designed to be 1 mm in diameter, as we know that we could maintain the self-renewal potential of hPSCs relatively well in this size in cell clump culture. Furthermore, patterned culture provides a location (address) and number for each individual cell spot and allows visual inspection and image acquisition of each colony with identification. It also introduces the concept of quality control of production units (spots) and a batch control by sampling a few spots for testing (S2B Fig).

### Differentiation potential and genetic stability of hPSCs cultured with SPM and rhVNT-N

**Fig 4:**
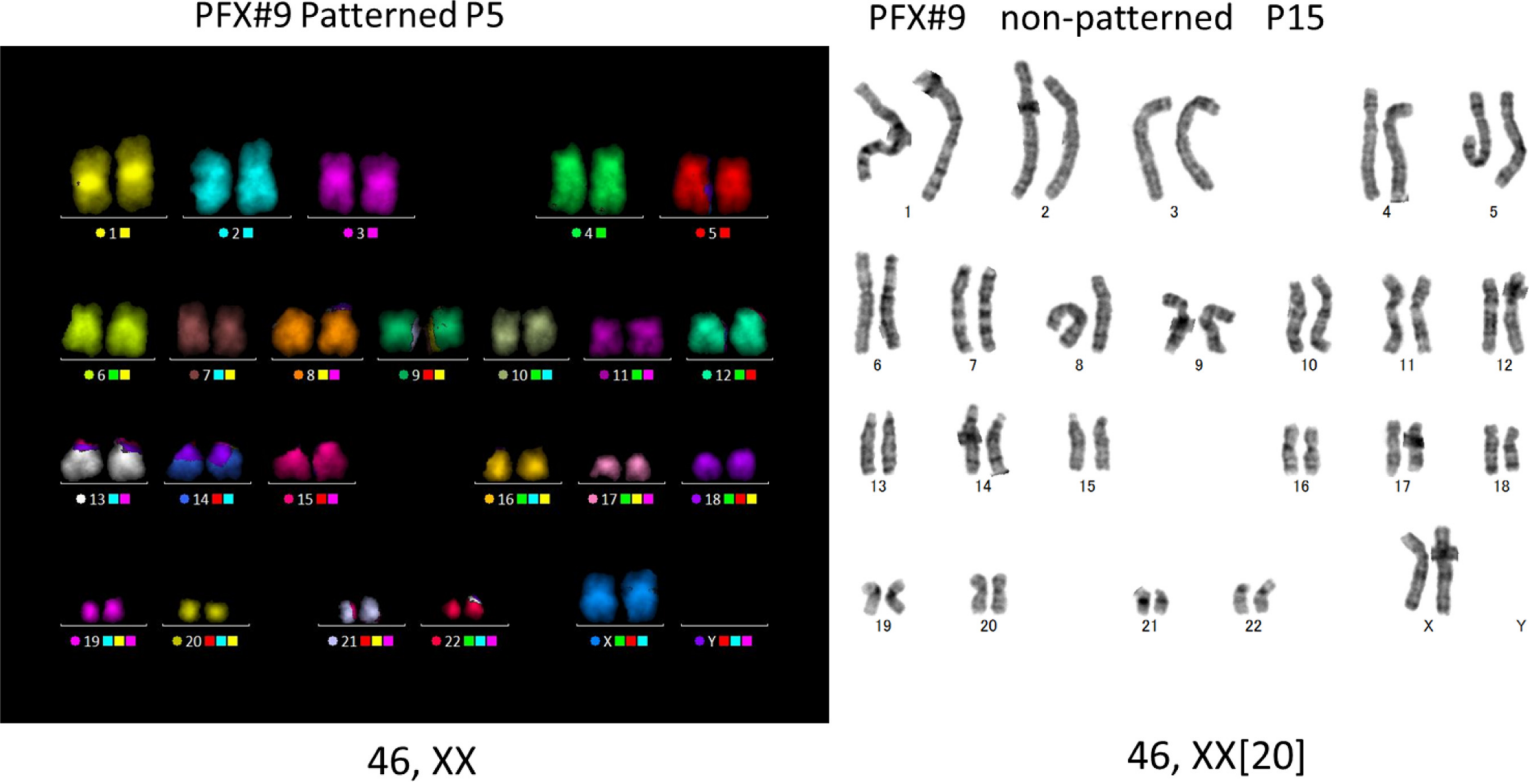
Karyotyping. Karyotype of PFX#9 maintained with SPM on rhVNT-N-coated dish (single cell patterning culture) was analyzed by mFISH at passage 5 (left). PFX#9 from single cell non-patterned culture underwent G-Band analysis at passage 15 (right).

hPSCs that were cultured with SPM on VTN-N-coated dishes as single cells and clumps were examined for their differentiation potential by forming embryoid bodies (EB). The three germ layer differentiation potentials of PFX#9 cells (Fig 3A, 3B, and 3C) or that of KhES-1 cells (S3 Fig) cultured in clumps, or on non-patterned dishes or on patterned dishes were examined via EB formation and found differentiation potential was not perturbed in these culture conditions. Karyotypes of PFX#9 or KhES-1 maintained as single cells with SPM on rhVNT-N-coated dishes were analyzed by mFISH or by G-Banding (Fig 4 and S5A and S5B Fig). Karyotypes of hPSCs in clumps were assessed by G-Band analysis (S5C and S5D Fig). We found normal karyotypes in all hPSCs that have been tested.

### Cryopreservation and thawing of hPSCs as single cells

**Fig 5:**
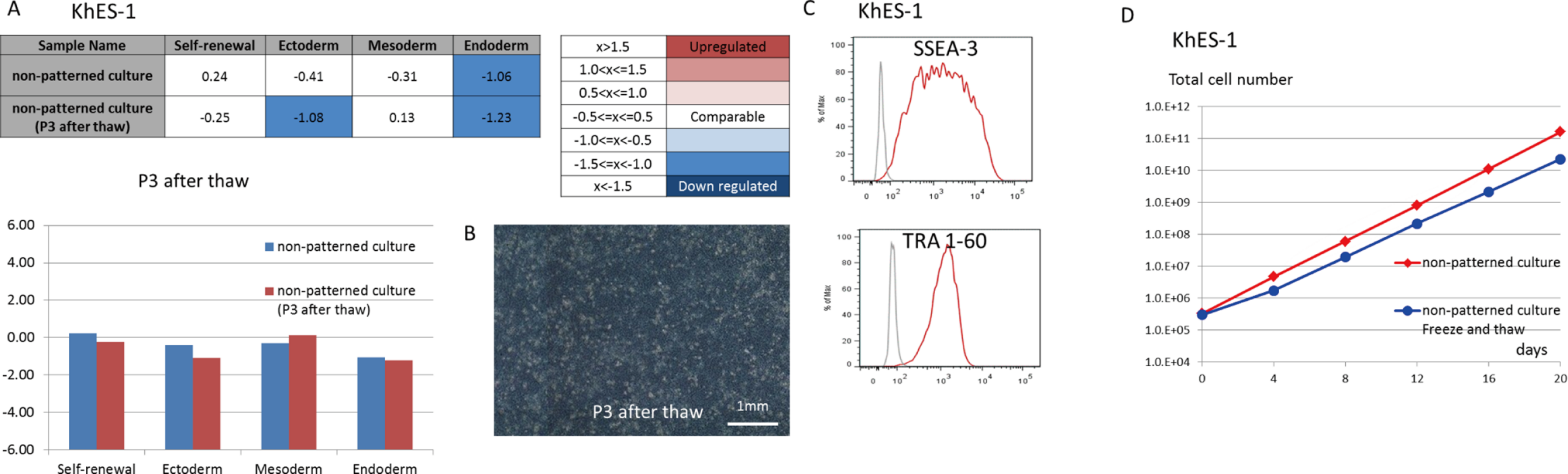
Cryopreservation and rapid thaw of KhES-1 cells grown in single cell non-patterned culture. (A) 1x10^6^ KhES-1 cells in single cell suspension from single cell non-patterned culture were cryopreserved with freezing medium, STEMCELL BANKER. The cells were thawed and cultured for 3 passages as single cells before examining the gene expression profile with TaqMan hPSC Scorecard. (B) Phase contrast image of KhES-1 cells cultured with SPM as single cells on rhVNT-N-coated dishes 3 passages after thaw. (C)Expression of SSEA-3 and TRA-1-60 on KhES-1 cells before and 5 passages after thaw was evaluated by flow cytometry. (D) Growth curve of KhES-1 in single cell non-patterned flat culture after thaw (blue line) was examined in comparison with cells in single cell non-patterned culture without cryopreservation (red line). The cryopreserved KhES-1 showed the same proliferation rate as the control in the second passage after thaw. Scores in Tables are visualized in bar graph below.

KhES-1 cells that were cultured with SPM in single cell suspensions were cryopreserved with chemically defined freezing medium (STEM-CELLBANKER). After thawing, they grew robustly in single cell suspension with SPM on VNT-N-coated non-patterned dishes. Gene expression profiles of cells 3 passages after thaw are shown in Fig 5A. KhES-1 cells maintained an undifferentiated state after the thaw. A photograph of KhES-1 cells 3 passages after thawing is shown in Fig 5B. Expression of self-renewal-related molecules SSEA-3 and TRA1-60 by KhES-1 5 passages after thawing was determined by flow cytometry (Fig 5C). Post-thaw cell growth of KhES-1 after the second passage was comparable to that seen before cryopreservation (Fig 5D). These data indicated that KhES-1 could be efficiently cryopreserved with freezing medium STEM-CELLBANKER and thawed and cultured with SPM as single cells on rhVNT-N-coated dishes.

## Discussion

In this report, we have shown that hPSCs can be cultured with a defined medium, SPM, on a very cost-effective ECM substrate (rhVNT-N) in the form of cell clumps or single cells (non-patterned or patterned). One of the features of SPM is that hPSCs cultured on feeders can be easily adapted to feeder-free conditions in the form of cell clumps or single cells without complicated sequential adaptation procedures. Such procedures may become an issue when chemically defined media and Vitronectin-N are used for cultivation and coating materials of dishes. SPM is also optimized to culture hPSCs in ECM-coated patterned culture dishes. SPM contains BSA produced in New Zealand, and it meets safety criteria as stated in the TSE guideline (EMA/410/01 rev.3) [15] and geographical risk assessment of BSE (GRB, European Food Safety Authority) [16], considerations for future clinical application. In September, 2014, consultations with MHRA in the UK for clinical application of BSA-containing medium supported our strategy for clinical application.

Photolithographic technology has been used for manufacturing patterned culture dishes. This technology can generate a patterned dish of any size and any shape with very sharp boundaries at micrometer scales. Such patterned dishes have been used for culturing mesenchymal stem cells [20]. But, coating ECM on patterned dishes is unique. This technology can reproducibly generate patterned dishes coated with ECM of any type including Laminin 521, Laminin 511 and Vitronectin-XF with even thickness and can avoid non-homogeneous coating. The latter sometimes occurs with ECM stamping using a mold or when a drop of ECM is placed directly on a dish. Micropatterned dishes with several shapes using the same photolithographic technology and the same PVA-base material BIOSURFINE-AWP are available commercially (Toyo Gosei Co. http://www.toyogosei.co.jp/eng/product/medical/).

A remaining concern for safety issues in clinical application of micropatterned dish is the possibility that PVA might elute into the culture medium and cause toxic effects to cells in culture. However, the growth rates of hPSCs in PVA-coated dish are comparable to those in non-coated dishes per culture area and no genetic abnormality was reported in several cell lines that have been tested for up to 20 passages. We believe that we will be able to prepare a toxicological test package for PVA prior to the application for clinical trial.

The passage of hPSCs as single cells was advantageous compared to passage via cell clumps (classical method). Some hPSC clumps include differentiated cells, and such differentiated clumps can attach to ECM after passage and proliferate. It is necessary to pick up and remove such differentiated clumps daily to maintain the culture’s undifferentiated state. We speculated that differentiated cells in single cell suspension could no longer attach to ECM with the same affinity, probably due to changes in their adhesion molecule expression profile and could be washed out at every medium change. In contrast, differentiated cells in clumps (differentiated clump) could remain attached to the ECM due to the strong affinity of the remaining undifferentiated cells for the ECM. To prove this speculation, a novel tracing method to mark cells early in differentiation was needed. Alternatively, we conducted comparison studies of gene expression profiles of cells from several culture conditions. Specifically, we used the qRT-PCR score card panel to examine the following: (a) cells from clumps that included differentiated cells (differentiated clumps), (b) cells from clumps lacking differentiated cells (undifferentiated clumps, after removal of differentiated clumps), (c) cells from single cell culture that were passaged from single cells for several passages, and (d) cells from single cell culture that were passaged from differentiated clumps (a). We found that cells from differentiated clumps most likely differentiated toward ectoderm among the three germ layers (S4A and S4B Fig), just like the first developmental event occurs in embryo after the implantation into uterus is ectodermal differentiation. However, cell populations ceased to demonstrate a potential for ectodermal differentiation after seeding in flat dishes in single cell suspensions, showing a comparable gene expression profile to undifferentiated clumps or undifferentiated single flat cell culture maintained as single flat cultures for extended periods (S4C and S4D Fig). Single cell passage also permitted quality control of cultured cells (examining viability and expression of surface molecules during passage and before/after cryopreservation) at the single cell level, which is an additional advantage of single cell culture over clump culture.

Patterning hPSCs in hundreds of round shaped spots provides several advantages over non-patterned single cell culture. For example, patterned culture supports controlled cell growth and the maintenance of the undifferentiated state of hPSCs by controlling the area for cell culture at around 1 mm in diameter, while cells cultured in a non-patterned dish grow unevenly and may lose the potential for pluripotency after many passages (Fig 2B, S1B and S2A Figs). It also facilitates the sampling of a few spots for quality control of a batch defined as one culture dish and monitoring the cell growth of every colony having designated address in a time course manner.

In conclusion, we have introduced a new hPSCs culture system, combining the use of a defined culture medium (SPM) and rhVNT-N-coated patterned dishes. This culture system provides a new method that can assure the quality of cultured hPSCs and the technological basis for the design of fully automated hPSCs mass closed culture system in the near future.

## Supporting Information

S1 Fig hPSCs culture with SPM on rhVNT-N coated dishes. (A) Phase contrast microscopic observation of KhES-1 cells under the indicated culture conditions at passage 15. (B) Expression of SSEA-3 and TRA-1-60 on KhES-1 determined by flow cytometry after culture under the indicated conditions at passage 15. (C) Phase contrast microscopic observation of cell cultures (clump, single cell non-patterned and single cell patterned) for ESC H9 cells and iPSC 201B7 cells. (D) Densities (cells/mm^2^) of KhES-1 cells in single cell non-patterned (non-patterned) or in single cell patterned (patterned) cultures were calculated by dividing harvested cell number by 962 mm^2^ (35-mm non-patterned culture dish) or dividing harvested cell number by 156 mm^2^ (total 199 spots of 1 mm diameter in 35-mm patterned culture dish). The result was obtained by scoring harvested cell numbers from 20 passages of the indicated culture and shown as a bar (mean) with error bar (standard deviation). The significance of difference between 2 groups: p = 3.6 x 10^−16^. Growth curve of KhES-1 cells (E), 201B7 cells (F) or H9 cells (G) in single cell non-patterned (non-patterned), single cell patterned (patterned) cultures or clump cultures. X-axis represents days of culture. PCSs in patterned culture or non-patterned culture were passaged every 4 days and in clump culture on feeder-free every 6 days and on feeder (SNL) every 5 days.
(TIF)

S2 Fig Quality control and sampling of hPSCs on rhVNT-N-coated dish in single cell passages. (A) Flow cytometric analysis of PFX#9 cells for the expression of SSEA-3 and TRA-1-60 cultured in non-patterned dishes. Gated population was sorted at passage 20 (left) and reanalyzed at passage 21 (middle) and 24 (right). (B) Patterned cell colony (spot) was removed by pipetting for cell sampling.
(TIF)

S3 Fig Differentiation potential of KhES-1 cells. Gene expression profiles of KhES-1 cells under the indicated culture conditions (clump, single cell non-patterned or single cell patterned) before (undifferentiated state) and after induction of differentiation via embryoid body (EB) formation. Average gene expression of self-renewal (undifferentiated state), ectoderm-, mesoderm- or endoderm-related genes is compared with reference standards of TaqMan hPSC Scorecard Panel (Life Technology). Scores in Table are visualized in bar graph below.
(TIF)

S4 Fig Differentiated cells in clumps after seeding on non-patterned dish as single cells. (A) a: H9 cells cultured in a clump on a VTN-N-coated dish started to differentiate. Cell clumps having morphology of differentiation are marked as red square, undifferentiated cell clump is marked as blue square. b: undifferentiated colonies after removing differentiated colonies. c: single cell flat non-patterned culture on VTN-N. (B) Gene expression profile of H9 with TaqMan hPSC Scorecard Panel to detect trend for differentiation of hPSCs in culture conditions stated in A. Relative gene expression of two representative genes for self-renewal, ectoderm, mesoderm or endoderm differentiation are presented as bar graph. (C) a: KhES-1 cells cultured in a clump on VTN-N coated dish started differentiation (indicated by white arrows). b: Undifferentiated colonies in clump culture after removing differentiated colonies. c: Non-patterned culture after 15 passages. d: Cells passaged from “a” were dispersed into single cells and seeded on Vitronectin-N coated non-patterned dish. (D) Gene expression profile of KhES-1 cells with TaqMan hPSC Scorecard Panel to detect trend for differentiation in hPSCs in culture conditions stated in “C”. Relative gene expression of two representative genes for self-renewal, ectoderm, mesoderm or endoderm differentiation are presented as bar graph.
(TIF)

S5 Fig Karyotype of cultured cells. (A) Karyotype of KhES-1 cells from patterned culture at passage 5 (P5) by mFISH. (B) Karyotype of KhES-1 from single cell non-patterned culture at P15 by G-Band analysis. (C) Karyotype of KhES-1 cells in clump culture at P22. (D) Karyotype of PFX#9 cells in clump culture at P25 by G-band analysis.
(TIF)

S1 Movie Patterned cultured of hPSCs. Cell proliferation of PFX#9 cultured with SPM on rhVTN-N-coated patterned dish was observed in a time lapse (up to 96 h).
(AVI)
