# Supplementary figures and images for: Controlled Growth and the Maintenance of Human Pluripotent Stem Cells by Cultivation with Defined Medium on Extracellular Matrix-Coated Micropatterned Dishes
Source: PLoS One. 2015 Jun 26;10(6):e0129855. doi: 10.1371/journal.pone.0129855 (PMC4483150; doi:10.1371/journal.pone.0129855)

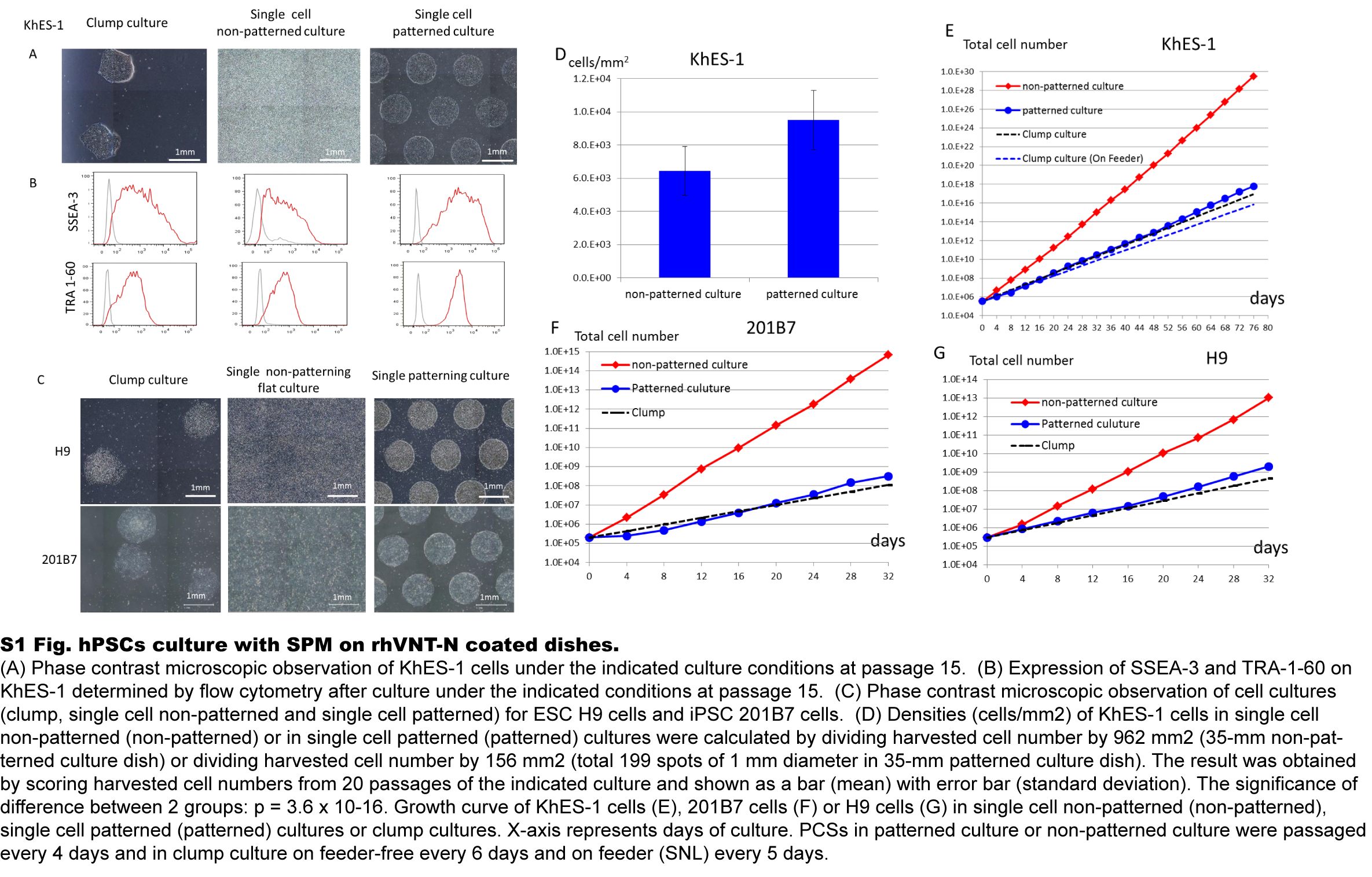

Supplement: S1 Fig — (A) Phase contrast microscopic observation of KhES-1 cells under the indicated culture conditions at passage 15. (B) Expression of SSEA-3 and TRA-1-60 on KhES-1 determined by flow cytometry after culture under the indicated conditions at passage 15. (C) Phase contrast microscopic observation of cell cultures (clump, single cell non-patterned and single cell patterned) for ESC H9 cells and iPSC 201B7 cells. (D) Densities (cells/mm2) of KhES-1 cells in single cell non-patterned (non-patterned) or in single cell patterned (patterned) cultures were calculated by dividing harvested cell number by 962 mm2 (35-mm non-patterned culture dish) or dividing harvested cell number by 156 mm2 (total 199 spots of 1 mm diameter in 35-mm patterned culture dish). The result was obtained by scoring harvested cell numbers from 20 passages of the indicated culture and shown as a bar (mean) with error bar (standard deviation). The significance of difference between 2 groups: p = 3.6 x 10^−16^. Growth curve of KhES-1 cells (E), 201B7 cells (F) or H9 cells (G) in single cell non-patterned (non-patterned), single cell patterned (patterned) cultures or clump cultures. X-axis represents days of culture. PCSs in patterned culture or non-patterned culture were passaged every 4 days and in clump culture on feeder-free every 6 days and on feeder (SNL) every 5 days. (TIF) [file pone.0129855.s001.tif]

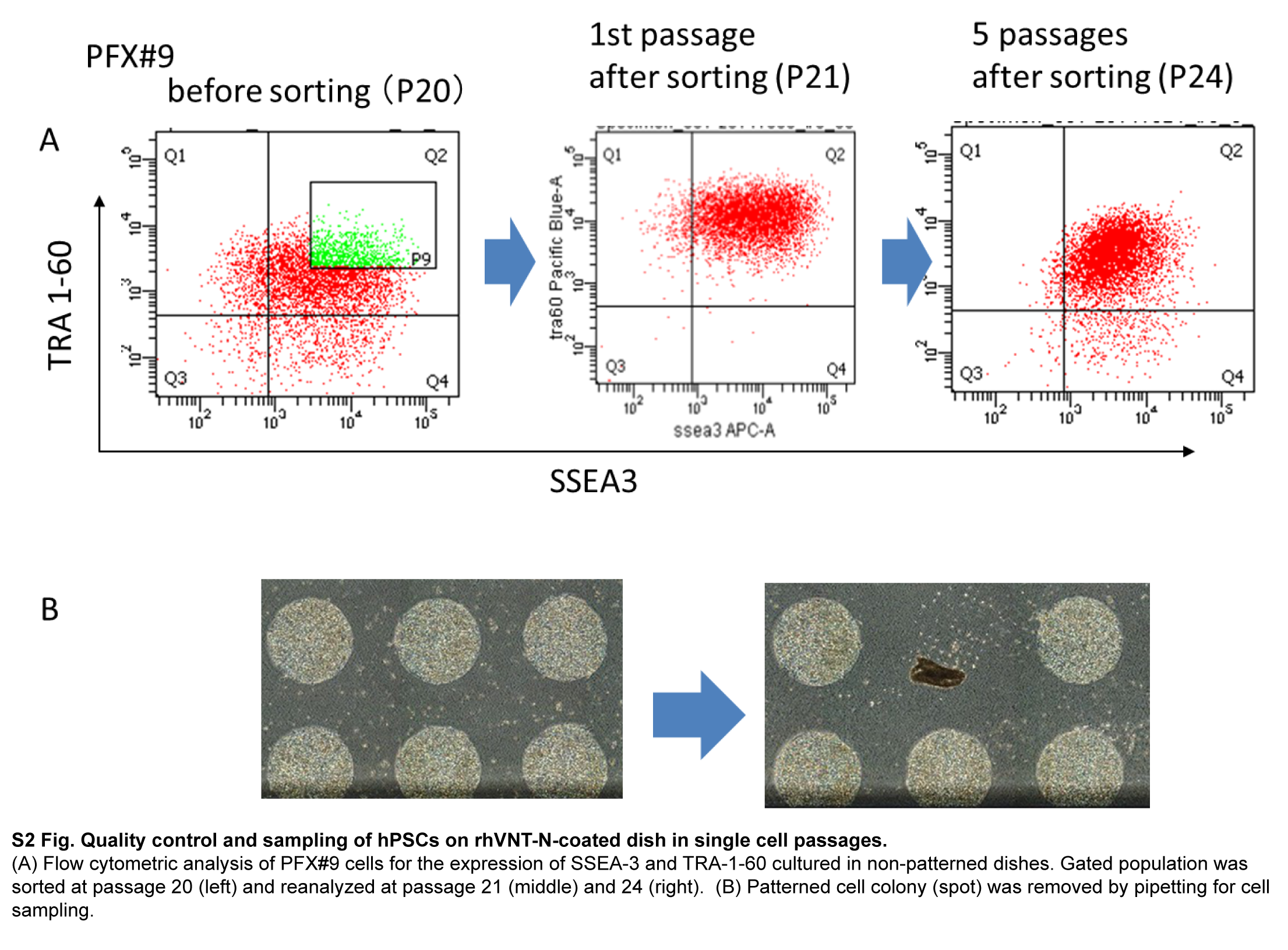

Supplement: S2 Fig — (A) Flow cytometric analysis of PFX#9 cells for the expression of SSEA-3 and TRA-1-60 cultured in non-patterned dishes. Gated population was sorted at passage 20 (left) and reanalyzed at passage 21 (middle) and 24 (right). (B) Patterned cell colony (spot) was removed by pipetting for cell sampling. (TIF) [file pone.0129855.s002.tif]

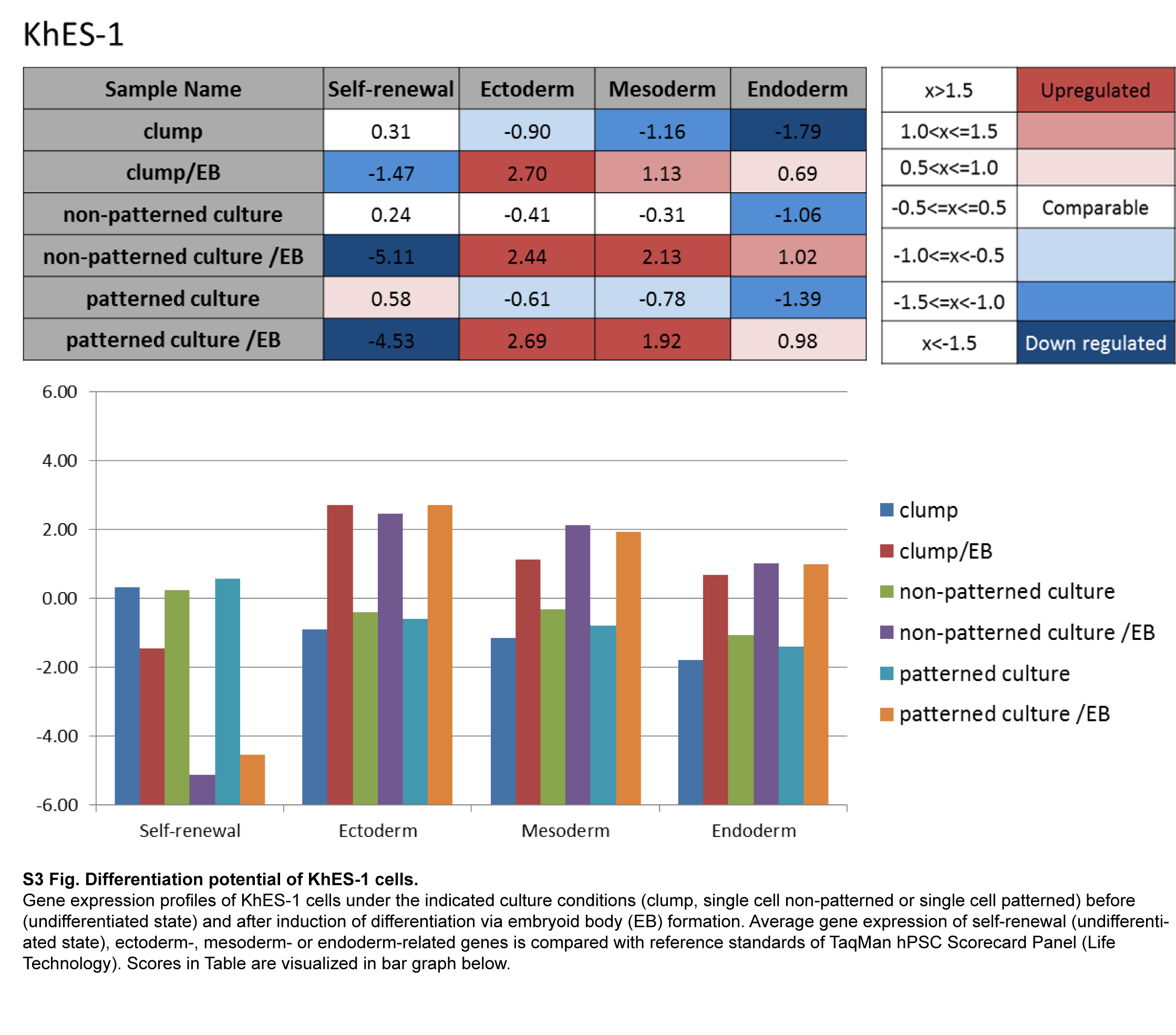

Supplement: S3 Fig — Gene expression profiles of KhES-1 cells under the indicated culture conditions (clump, single cell non-patterned or single cell patterned) before (undifferentiated state) and after induction of differentiation via embryoid body (EB) formation. Average gene expression of self-renewal (undifferentiated state), ectoderm-, mesoderm- or endoderm-related genes is compared with reference standards of TaqMan hPSC Scorecard Panel (Life Technology). Scores in Table are visualized in bar graph below. (TIF) [file pone.0129855.s003.tif]

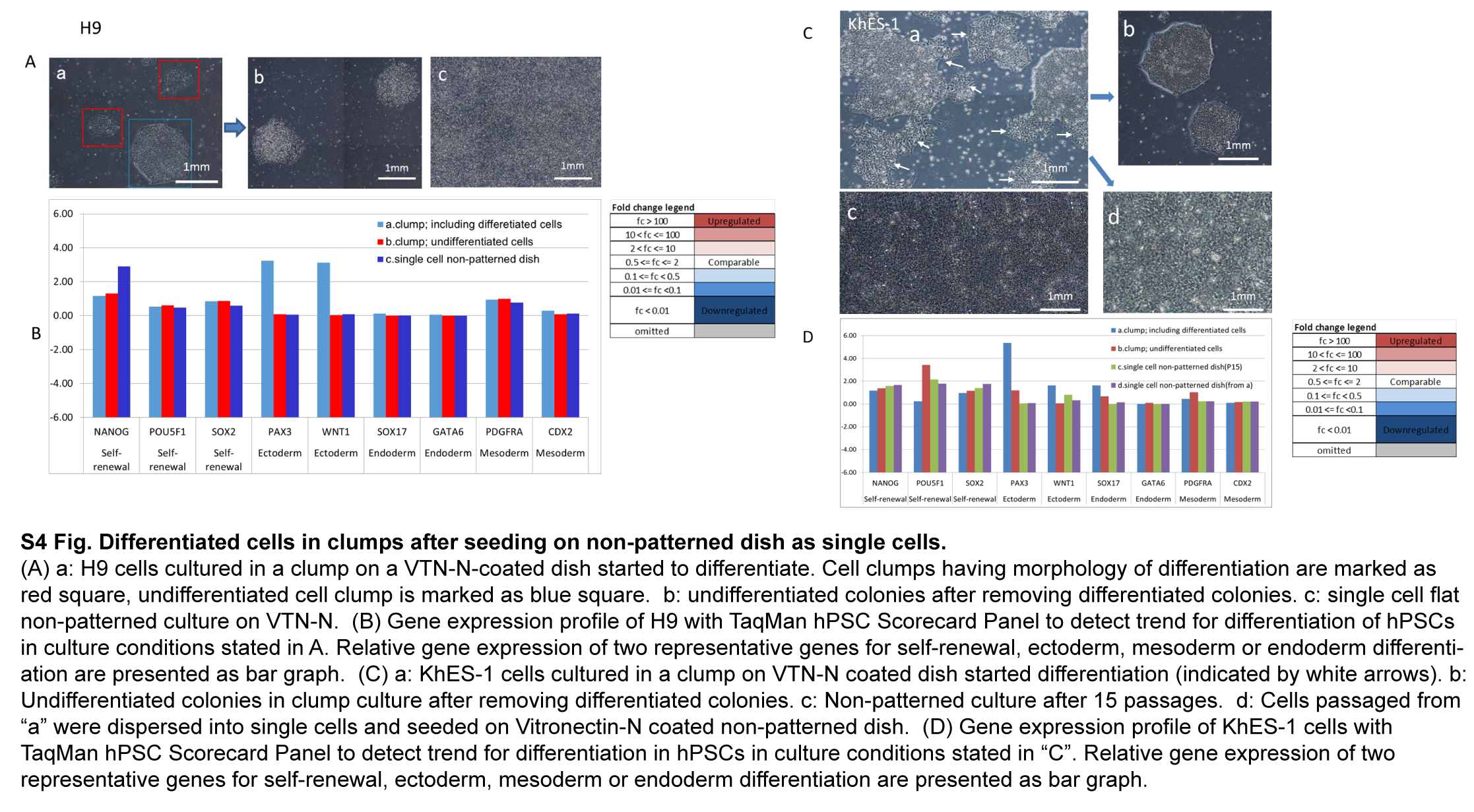

Supplement: S4 Fig — (A) a: H9 cells cultured in a clump on a VTN-N-coated dish started to differentiate. Cell clumps having morphology of differentiation are marked as red square, undifferentiated cell clump is marked as blue square. b: undifferentiated colonies after removing differentiated colonies. c: single cell flat non-patterned culture on VTN-N. (B) Gene expression profile of H9 with TaqMan hPSC Scorecard Panel to detect trend for differentiation of hPSCs in culture conditions stated in A. Relative gene expression of two representative genes for self-renewal, ectoderm, mesoderm or endoderm differentiation are presented as bar graph. (C) a: KhES-1 cells cultured in a clump on VTN-N coated dish started differentiation (indicated by white arrows). b: Undifferentiated colonies in clump culture after removing differentiated colonies. c: Non-patterned culture after 15 passages. d: Cells passaged from “a” were dispersed into single cells and seeded on Vitronectin-N coated non-patterned dish. (D) Gene expression profile of KhES-1 cells with TaqMan hPSC Scorecard Panel to detect trend for differentiation in hPSCs in culture conditions stated in “C”. Relative gene expression of two representative genes for self-renewal, ectoderm, mesoderm or endoderm differentiation are presented as bar graph. (TIF) [file pone.0129855.s004.tif]

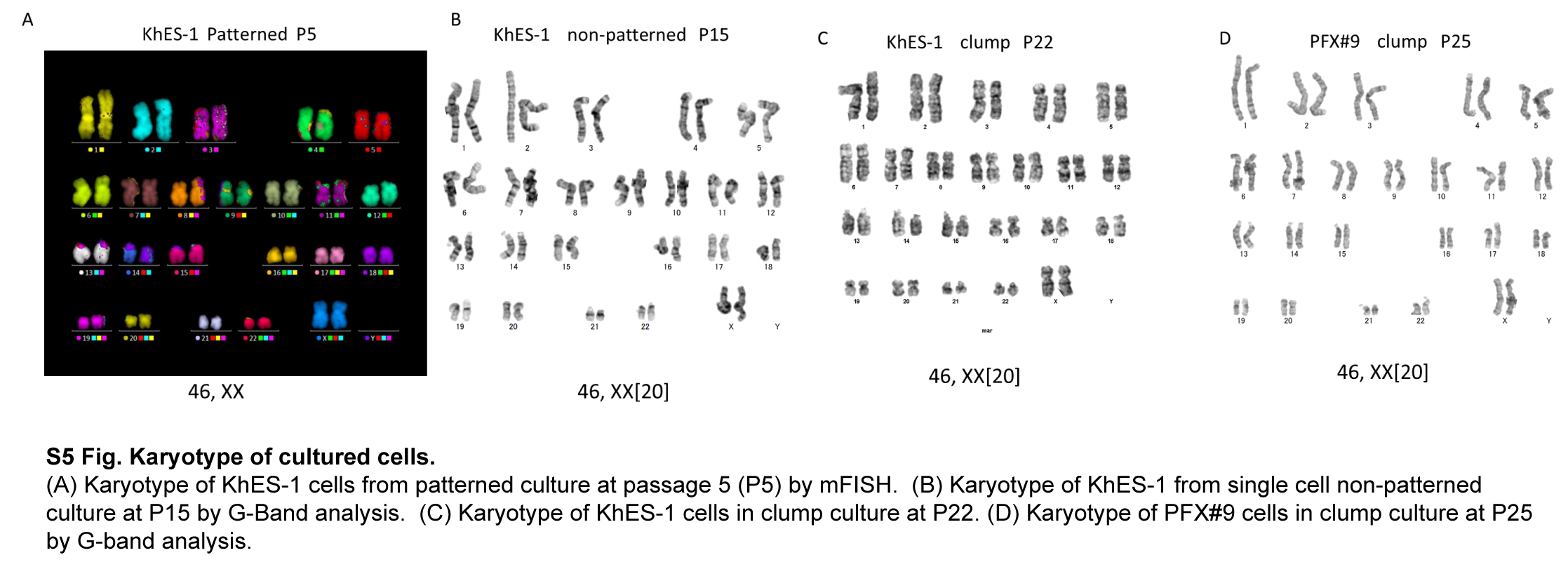

Supplement: S5 Fig — (A) Karyotype of KhES-1 cells from patterned culture at passage 5 (P5) by mFISH. (B) Karyotype of KhES-1 from single cell non-patterned culture at P15 by G-Band analysis. (C) Karyotype of KhES-1 cells in clump culture at P22. (D) Karyotype of PFX#9 cells in clump culture at P25 by G-band analysis. (TIF) [file pone.0129855.s005.tif]
